# COVID-19: Myths and Reality

**DOI:** 10.1134/S0006297921070026

**Published:** 2021-07-08

**Authors:** Larisa V. Kordyukova, Andrey V. Shanko

**Affiliations:** 1 Belozersky Institute of Physicochemical Biology, Lomonosov Moscow State University, 119991 Moscow, Russia; 2 FORT LLC, R&D Department, 119435 Moscow, Russia; 3 Ivanovsky Institute of Virology, Gamaleya Federal Research Center for Epidemiology and Microbiology, 123098 Moscow, Russia

**Keywords:** COVID‑19, SARS‑CoV‑2, S protein, structure, influenza virus, hemagglutinin, vaccines

## Abstract

COVID‑19, a new human respiratory disease that has killed nearly 3 million people in a year since the start of the pandemic, is a global public health challenge. Its infectious agent, SARS‑CoV‑2, differs from other coronaviruses in a number of structural features that make this virus more pathogenic and transmissible. In this review, we discuss some important characteristics of the main SARS‑CoV‑2 surface antigen, the spike (S) protein, such as (i) ability of the receptor-binding domain (RBD) to switch between the “standing-up” position (open pre-fusion conformation) for receptor binding and the “lying-down” position (closed pre-fusion conformation) for immune system evasion; (ii) advantage of a high binding affinity of the RBD open conformation to the human angiotensin-converting enzyme 2 (ACE2) receptor for efficient cell entry; and (iii) S protein preliminary activation by the intracellular furin-like proteases for facilitation of the virus spreading across different cell types. We describe interactions between the S protein and cellular receptors, co-receptors, and antagonists, as well as a hypothetical mechanism of the homotrimeric spike structure destabilization that triggers the fusion of the viral envelope with the cell membrane at physiological pH and mediates the viral nucleocapsid entry into the cytoplasm. The transition of the S protein pre-fusion conformation to the post-fusion one on the surface of virions after their treatment with some reagents, such as β-propiolactone, is essential, especially in relation to the vaccine production. We also compare the COVID‑19 pathogenesis with that of severe outbreaks of “avian” influenza caused by the A/H5 and A/H7 highly pathogenic viruses and discuss the structural similarities between the SARS‑CoV‑2 S protein and hemagglutinins of those highly pathogenic strains. Finally, we touch on the prospective and currently used COVID‑19 antiviral and anti-pathogenetic therapeutics, as well as recently approved conventional and innovative COVID‑19 vaccines and their molecular and immunological features.

## INTRODUCTION

The outbreak of a disease with the symptoms of atypical pneumonia first started in Wuhan (Hubei province, China) in winter 2019. The causative agent was later identified as a novel human coronavirus (2019‑nCoV, 2019 novel coronavirus), or SARS‑CoV‑2 (severe acute respiratory syndrome coronavirus 2). In February 2020, World Health Organization named the disease COVID‑19 (COronaVIrus Disease 2019). The outbreak has become global and was declared a pandemic. The majority of patients infected with SARS‑CoV‑2 exhibit mild or moderate symptoms, which disappear after 6-10 days. However, almost 20% patients develop serious complications, including atypical bilateral pneumonia and acute respiratory distress syndrome associated with high lethality [1]. As of April 5, 2021, the number of deaths resulting from COVID‑19 has reached 2.85 million out of 131 million people diagnosed (~1-3% death rate according to the World Health Organization; https://covid19.who.int/) [2]. For comparison, seasonal flu causes 250,000-500,000 deaths annually [3]; the global mortality during the H1N1 influenza pandemic in 2009 was 151,700 to 575,400 deaths [4].

Enveloped RNA viruses of the Coronaviridae family were first isolated as human infectious agents in 1960s. Four representatives of coronaviruses infecting humans (HCoV‑NL63, HCoV‑229E, HCoV‑OC43, and HKU1) cause mainly mild upper respiratory tract infections, that can be more serious in small children and elderly [5]. Deaths associated with the coronavirus infections were reported much later. The highly pathogenic SARS‑CoV virus (severe acute respiratory syndrome coronavirus) was identified as a cause of atypical pneumonia outbreak in 2002; in 2012, it was found that the newly classified disease, Middle East respiratory syndrome, is induced by MERS‑CoV (Middle East Respiratory Syndrome coronavirus). The fatality in the atypical pneumonia and MERS infection was 9.5 and 34.4%, respectively [6]. Luckily, the spread of both these diseases has been limited to relatively small geographic areas.

In this review, we discuss the classification of coronaviruses, the features that distinguish SARS‑CoV‑2 from other human coronaviruses, and major characteristics of the structural components of this virus with special emphasis on the spike protein (S protein). As the main immunity target, S protein is of particular interest for the vaccine development [7]. Below, we will discuss interactions of this protein with cellular receptors, co-receptors, and antagonists, as well as the mechanism of membrane destabilization that initiates the fusion of the viral envelope with the cell membrane at physiological pH and facilitates penetration of the viral genome into the cytoplasm for its further replication.

Severe COVID‑19 cases are characterized by a number of pathogenetic factors similar to those observed during severe influenza illness. Influenza is caused by various representatives of Orthomyxoviridae, another family of enveloped viruses. Some of them, such as 2009 pandemic influenza A/H1N1 virus strains and the highly pathogenic avian influenza A/H5N1 and A/H7N9 viruses result in much more drastic effects on human health compared to the strains causing seasonal flu. There are some similarities between the structural properties of surface glycoprotein hemagglutinin (HA) of highly pathogenic avian influenza viruses and SARS‑CoV‑2 S protein, as well as between the clinical symptoms of these diseases (as discussed below).

Finally, we discuss the problems associated with suppression of the viral infection and list the strategies used for the COVID‑19 therapy, as well as present currently existing COVID‑19 vaccines and discuss the principles underlying the action of these classic and innovative vaccines.

## GENERAL CHARACTERISTIC OF CORONAVIRUSES

The viruses belonging to Coronaviridae family (order Nidovirales) infect various hosts, including birds and mammals. This family consists of two subfamilies: all human-infecting species belong to the Orthocoronavirinae subfamily that includes four genera: *Alphacoronavirus*, *Betacoronavirus*, *Gammacoronavirus*, *Deltacoronavirus* (https://talk.ictvonline.org/taxonomy/). The *Betacoronavirus* genus includes four subgenera (or lineages, according to the previous classification): *Embecovirus* (lineage A), *Sarbecovirus* (lineage B), *Merbecovirus* (lineage C), and *Nobecovirus* (lineage D) [8]. Viruses HCoV‑229E, HCoV‑NL63 (genus *Alphacoronavirus*), HCoV‑OC43, and HCoV‑HKU1 (genus *Betacoronavirus*) cause moderate respiratory illnesses in humans. SARS‑CoV‑2 belongs to the *Betacoronavirus* genus, *Sarbecovirus* subgenus. The closely related SARS‑CoV (SARS‑CoV‑1) belongs to the same subgenus, while MERS‑CoV was attributed to the subgenus *Merbecovirus*.

**Fig. 1.**
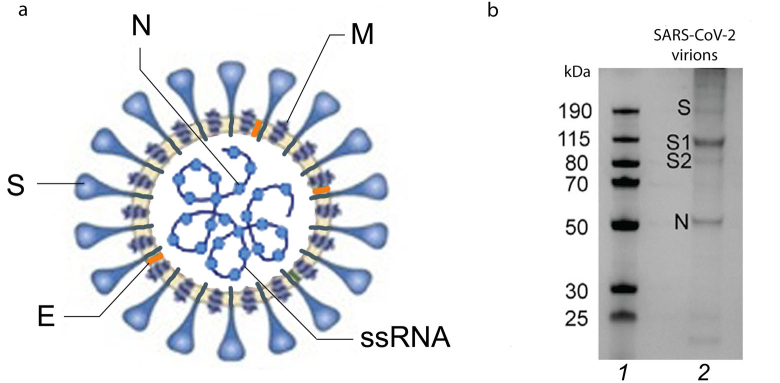
Structure of SARS‑CoV‑2 virion and its structural proteins. a) SARS‑CoV‑2 virion with its structural proteins (S, N, M, E) and genomic single stranded (+)RNA (+ssRNA): S, spike protein; E, envelope protein; M, membrane protein; N, nucleocapsid protein. b) SDS-PAGE of purified SARS‑CoV‑2 virions. Lanes: *1*) molecular weight markers; *2*) major structural proteins (S, its subunits S1 and S2 after proteolytic activation, and N). Adapted with permission from Yao et al. [9]. Copyright Elsevier, 2020.

Spherical or slightly polymorphic virions of coronaviruses are 80-120 nm in diameter. They are surrounded by the lipoprotein envelope and have spikes on the surface [9, 10] that resemble a royal crown (*corona* in Latin); hence the name coronavirus (Fig. 1a). Morphologically, the virions of coronaviruses are similar to the influenza virions, except they carry only one type of spikes represented by the homotrimers of S protein [11, 12], while virions of the influenza A and B viruses have two types of spikes – HA homotrimers and neuraminidase (NA) homotetramers [13, 14].

**The genome** of coronaviruses consists of a single-stranded positive-sense RNA molecule (+ssRNA) of ~27 to 32 Kb (29.3 Kb in SARS‑CoV‑2). This is the largest and very well-organized genome among all RNA viruses (for comparison, influenza virus genome is 13.5 Kb). The coronaviral RNA encodes 28-29 proteins, including 4 or 5 structural proteins. Human viruses HCoV‑229E, HCoV‑NL63, SARS‑CoV, SARS‑CoV‑2, and MERS‑CoV have four structural proteins: spike protein (S); envelope protein (E); membrane protein (M); and nucleocapsid protein (N) (Fig. 1a). HCoV‑OC43 and HCoV‑HKU1 virions have the fifth structural protein in their composition (hemagglutinin esterase, HE).

**S protein of coronaviruses with a molecular mass of ~175 kDa** [15] is a type I membrane protein consisting of a large glycosylated N-terminal ectodomain, one transmembrane (TM) domain, and C-terminal intraviral (or cytoplasmic, CT) domain [11, 12]. S protein binds to receptors on the cell surface and plays an important role in the tissue tropism of the virus. It also mediates the fusion of the viral membrane with the host cell membrane, ensuring translocation of the viral genome into the cell cytoplasm. In addition, it participates in the virion assembly. Electrophoresis of the native virion proteins run under denaturing conditions demonstrated the presence of a fraction of unprocessed (inactive) S protein, while a larger fraction is represented as two S protein subunits (S1 and S2) (Fig. 1b). S protein epitopes are the main antigens that stimulate formation of neutralizing antibodies and serve as targets of cytotoxic lymphocytes.

**M protein (membrane protein)** (~25-30 kDa) contains 3 TM domains [16, 17]. It has a small N-terminal glycosylated ectodomain and a larger C-terminal endodomain, which extends 6-8 nm inside the viral particle [17, 18]. M protein determines the shape of the virion and ensures formation of contacts between various structural proteins during virion assembly [16, 19]. Electron cryotomography (cryo-ET) studies have demonstrated that the M protein exists as a dimer and can assume two different conformations. It contributes to the membrane curvature, which is important during formation of progeny virions.

**N protein (nucleocapsid protein)** (~46-48 kDa) is associated with the viral (+)RNA and forms the nucleocapsid. It participates in the regulation of viral RNA synthesis and interacts with the M protein during the virus budding [16]. Several antigenic epitopes have been predicted in the N protein, making it one of the important coronaviral antigens [20].

**E protein (envelope protein)** (~8-12 kDa) is a type III transmembrane protein, which has a small N-terminal ectodomain (~16 amino acid residues, aa), one TM domain, and a CT domain consisting of ~37-50 aa. Functionally, this protein is similar to the M2 protein of the influenza virus: it exhibits the porin activity (forms a pentameric ion channel in the lipid membrane) and participates in the assembly and budding of viral particles [16]. Three conserved cysteine residues in the E protein are S-acylated, which is important for the virion assembly [21-23]. The E and M proteins affect the intracellular transport, proteolysis, and N-glycosylation of S protein [24].

**HE protein (hemagglutinin esterase)** has been found only in some β-coronaviruses, such as human HCoV‑OC43 and HKU1, and avian viruses of the *Deltacoronavirus* genus. A hemagglutinin domain of the HE protein binds to the neuraminic acid at the surface of the host cell and, likely, facilitates initial virus adsorption on the membrane, while esterase cleaves acetyl groups from the neuraminic acid. The genes coding for the HE proteins in coronaviruses are homologous to the genes coding for the hemagglutinin-esterase-fusion (HEF) glycoprotein of the influenza C virus.

The genome of SARS‑CoV‑2 codes for 16 non-structural proteins (nsp1-16) and 8 accessory proteins involved in the biogenesis of new viral particles [25]. Among them are RNA-dependent RNA polymerase (RdRp) catalyzing replication of the viral RNA, and two proteases – PLpro (papain-like cysteine protease) and 3CLpro/Mpro (chymotrypsin-like protease/main protease) responsible for the autolytic cleavage of viral polyproteins into the functional fragments.

## EVOLUTION AND LIFE CYCLE OF CORONAVIRUSES

**Multiple alignment** of nucleotide sequences from different coronaviruses shows that the closest relatives of SARS‑CoV‑2 are viruses infecting bats. However, infection of humans directly by the bat virus seems highly unlikely because the binding of the virus to the human angiotensin-converting enzyme 2 (ACE2) receptor is very weak [26]. Which intermediate host is involved in the zoonotic introduction of the novel coronavirus into the human population still remains debatable [27]. Some phylogenetic studies suggest pangolins as possible intermediate hosts. In particular, it was shown that the receptor-binding motif (RBM) of the SARS‑CoV‑2 S protein could be obtained by recombination with the pangolin virus [28, 29].

Unprecedented analysis of more than 200,000 SARS‑CoV‑2 whole genomic sequences revealed several mutations, among numerous purifying selection mutations, that could be explained by the positive selection pressure, in particular, 614G substitution in the S protein and several substitutions in the nucleocapsid protein (e.g., 203K) [30]. It was suggested that multiple changes in the N protein were important for the SARS‑CoV‑2 adaptation to humans [30].

The bioinformatics data on the percentages of intrinsic disorder in the N and M proteins from different coronaviruses are of particular interest [31]. The authors demonstrated that SARS‑CoV‑2 has a uniquely “rigid” (consisting of highly ordered proteins) protective coat, which might be a reason for the high stability of SARS‑CoV‑2 virions in saliva and other body fluids and in the environment [31].

**The life cycle of coronaviruses** begins from the virus entry into the cell. Two possible pathways have been suggested: (i) viral membrane fusion with the cell plasma membrane (which appears to be the main pathway) and (ii) endocytosis followed by the viral membrane fusion with the endosomal membrane. In both cases, S protein is involved in both interaction with the receptor and membrane fusion.

In the influenza virus, the protein responsible for virus entry into the cells is HA, which is a type I glycoprotein (similar to coronaviral S protein). HA binds to the cell surface proteins and lipids with exposed sialic acids. The membrane fusion requires the rearrangement of HA molecules that are induced in the cell endolysosomes at acidic pH. On the contrary, in the case of beta-coronaviruses, the fusion of the membranes mainly occurs at physiological (neutral) pH, and therefore, destabilization and rearrangement of the SARS‑CoV‑2 S protein should be triggered by some other factors. Below, we describe individual steps initiating this process and hypothetical mechanism of S protein destabilization.

Replication of coronaviral RNA takes place in specialized perinuclear structures, in particular, double-membrane vesicles containing double-stranded RNA molecules (according to cryo-ET) [9, 32]. Two types of the ribonucleoprotein (RNP) complexes have been detected in infected cells: spherical structures arranged in an “eggs in a nest” hexagonal assembly (one sphere in the center surrounded by six spheres) or as a pyramid (tetrahedron) composed of four spheres [9]. Statistical analysis revealed that the hexagonal and tetrahedral types of assembly corresponded to the spherical and ellipsoidal virions, respectively. Individual spherical structured were connected by thin thread-like structures. This type of arrangement allows the packing of an unusually large coronavirus genome into the inner volume of a virion with a diameter of 80-100 nm [9, 32] while preserving its high structural flexibility.

The N protein and non-structural coronavirus proteins are synthesized in the cytoplasm, while proteins of the viral membrane are synthesized on the endoplasmic reticulum membrane. It is likely that the S protein trimers assembled in the luminal cisternae participate in the organization of the budding sites in the endoplasmic-reticulum-Golgi intermediate compartment (ERGIC) [32]. The progeny virions bud from the ERGIC cisternae and are transported to the extracellular medium via exocytosis.

**Receptor recognition** is the first step of viral infection and a key determinant of the tropism of host cells and tissues. ACE2 is the main receptor for both SARS‑CoV‑2 and closely related SARS-CoV. ACE2 is a type I transmembrane protein and a dipeptidyl carboxypeptidase (EC 3.4.17.23). It is composed of approx. 805 aa and contains one zinc-binding domain [33]. The full-size ACE2 molecule includes the N-terminal peptidase domain and the C-terminal collectrin-like domain including the α-helical TM domain and the CT domain (~40 aa).

SARS‑CoV‑2 binds to ACE2 with the affinity that is 10 to 20 times higher than the affinity of SARS‑CoV [11, 34]. The peptidase domain plays the role of receptor for coronaviruses, but the binding does not require the peptidase activity [33]. ACE2 is predominately expressed in the lungs, heart, kidneys, ovaries, and gastrointestinal tract. In the lungs, ACE2 has been found in the type II alveolar epithelial cells, bronchial epithelium, and vascular endothelium. Based on the available structural information, it was suggested that the ACE2 alleles rs73635825 (S19P) and rs143936283 (E329G) could provide the organism resistance to the SARS‑CoV‑2 infection [35].

**Physiological role of ACE2** involves transformation of angiotensin I into angiotensin (1-9) and angiotensin II into angiotensin (1-7). Angiotensin (1-9) binds to the Mas receptor, which results in the vasodilatory and anti-inflammatory effects. This activity is opposite to the function of ACE, which transforms angiotensin I into angiotensin II. The vasoconstrictive and pro-inflammatory effects of angiotensin II are mediated by the type I angiotensin II receptor [33]. It is possible that virus binding to ACE2 shifts the equilibrium towards angiotensin II accumulation and, consequently, vasoconstriction and development of inflammatory reactions. These processes could facilitate thrombosis, although exact mechanisms of thrombus formation in the vessels are still poorly understood.

## STRUCTURAL STUDIES OF SARS‑CoV‑2 S PROTEIN

**Fig. 2.**
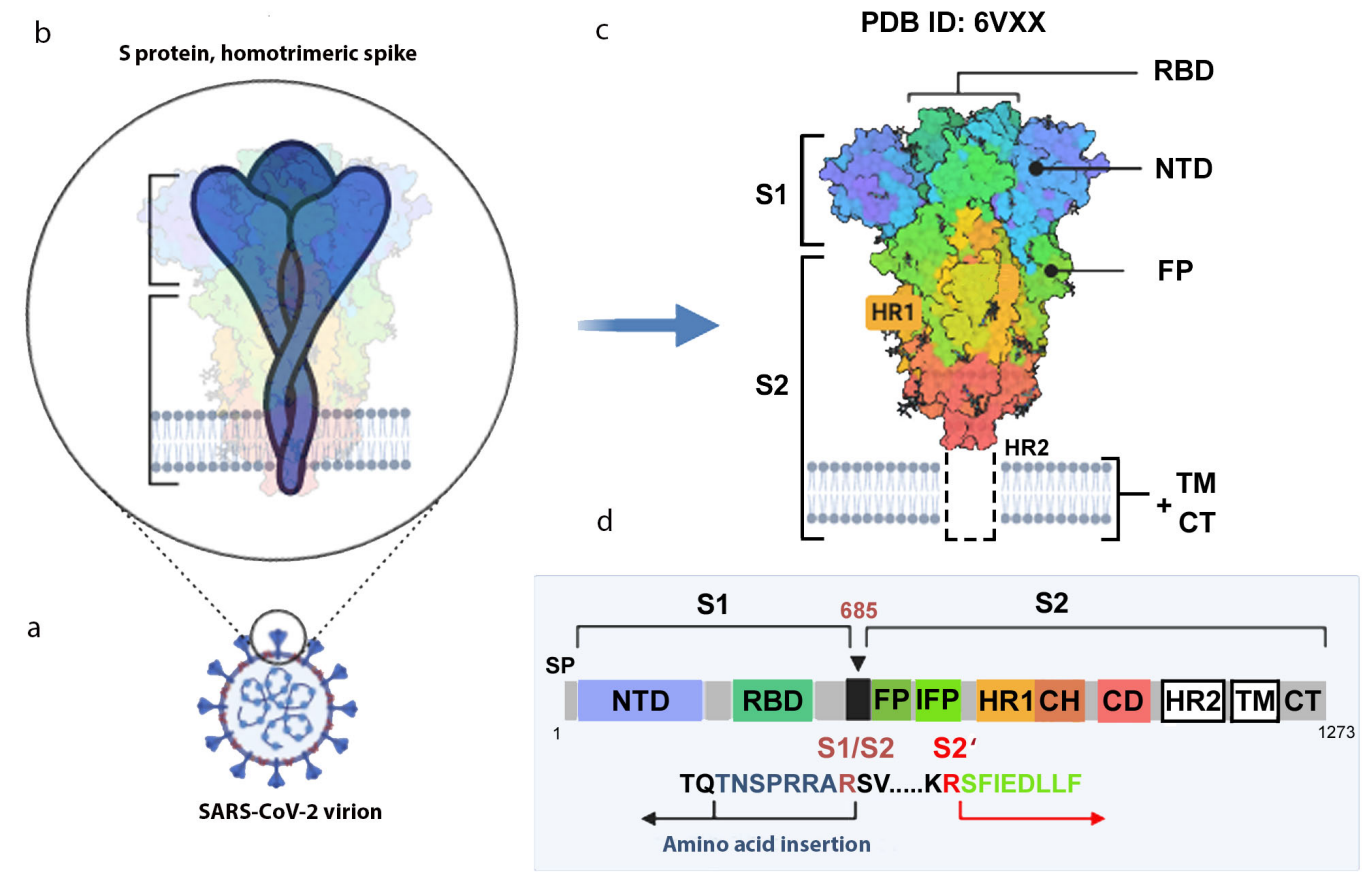
S protein structure. a) Virion with S protein spikes; b) homotrimeric spike anchored in the virion membrane; c) 3D-structure of the spike closed conformation (cryo-EM data, PDB ID 6VXX [12]); d) S protein monomer with indicated functional domains and proteolytic activation sites. S1 and S2, S protein subunits; S1/S2, furin cleavage site; SP, signaling peptide; NTD, N-terminal domain; RBD, receptor-binding domain; FP, fusion peptide; IFP, inner fusion peptide emerging after the S2 subunit cleavage at the S2′ site [36]; HR1 and HR2 (heptad repeats 1 and 2), specialized repeats in the amino acid sequence; TM, transmembrane domain; CT, cytoplasmic domain. The image was created using the BioRender.com pattern.

SARS‑CoV‑2 S protein forms homotrimers on the virion surface (Fig. 2, a-c). Each protein monomer is composed of 1273 aa (UniProt ID P0DTC2). After proteolytic cleavage of the signaling peptide (SP), the protein forms two functional subunits, S1 (aa 13-685) and S2 (aa 686-1273).

The distal S1 subunit facilitates stabilization of the pre-fusion state of the S2-subunit anchored in the virion membrane. S1 is responsible for the receptor binding, and S2 is responsible for the fusion. S1 consists of the *N*-terminal domain (NTD) and the C-terminal domain (CTD), also known as the receptor-binding domain (RBD, aa 319-541). The NTD has a topology of human galectins (galactose-binding lectins) [29]. The RBD contains two subdomains: the core (5-stranded antiparallel β-sheet) and the RBM itself (aa 437-508), the latter determines the specificity of protein binding. It was shown that the Gly-Val-Glu-Gly motif (aa 482-485) within the RBM ensures more efficient contact between the SARS‑CoV‑2 RBD and ACE2 (in comparison with SARS-CoV) [37].

**Protease-sensitive activation sites in S protein.** To ensure virus entry into the cell, S protein must be activated via proteolytic cleavage into S1 and S2 subunits that remain non-covalently bound in coronaviruses. The most striking variation of the amino acid sequence in the SARS‑CoV‑2 S protein that distinguishes it from its “precursor” (bat coronavirus BetaCoV/RaTG13/2013) and SARS-CoV is an insertion of positively charged amino acid residues at the S1/S2 site (Fig. 2d) [12, 29, 36]. Instead of a single arginine residue, the site contains the (682)R-R-A-R(685) ↓ sequence recognized by the furin-like proteases located in the Golgi apparatus [12, 36]. As cleavage by furin proteases occurs during the protein biosynthesis in the cell, the progeny SARS‑CoV‑2 virions receive pre-activated S protein, although a fraction of the S protein molecules in the virions remains intact (as demonstrated by electrophoresis, Fig. 1b) and, therefore, inactive. This significantly enhances the pathogenic potential of the virus, which acquires the ability to infect different types of cells in an organism beside the respiratory epithelium containing large amounts of extracellular trypsin-like proteases.

Considering that furin is highly expressed in the lungs, enveloped viruses infecting the airways could use this convertase to activate their surface glycoproteins. The furin cleavage site R-X-R/K-R has been found between the HA1 and HA2 subunits in the highly pathogenic strains of the avian influenza A/H5 and A/H7 subtype viruses [38]. The polybasic proteolytic sites were also identified in the surface glycoproteins of some Paramyxoviridae viruses capable of infecting humans [measles virus, mumps virus, respiratory syncytial virus (RSV)], pathogenic Newcastle disease virus (NDV); Ebola and Marburg viruses, yellow fever virus, HIV‑1, and a number of viruses of the Herpesviridae family dangerous for humans [38, 39].

After binding to the ACE2 receptor, all coronaviruses are cleaved at the inner S2′ site located immediately before the inner fusion peptide (IFP) sequence S-F-I-E-D-L-L-F [29, 36] (Fig. 2d). The cleavage at the S2′ site serves as a signal for the irreversible conformational changes in the S protein that promote the membrane fusion. S protein is cleaved at the S2’ site mainly by the membrane serine proteinase TMPRSS2 (transmembrane serine protease 2). Other cellular proteases, including cathepsins B and L (endosomal cysteine proteases), furin, and elastase, can also activate S protein. However, the activity of TMPRSS2 is considered strictly necessary for the spreading of SARS‑CoV‑2. The essential role of the TMPRSS2 in the influenza A virus pathogenesis was reported previously [40].

**Posttranslational modifications.** The surface of the homotrimeric spike is heavily glycosylated [9, 11, 12, 41-43]. Twenty-two N-glycosylation sites have been mapped in the SARS‑CoV‑2 S protein (66 sites in the homotrimer) that can carry extended carbohydrate chains. The two existing O-glycosylation sites (T323/S325) were found to be unmodified in 99% of the native protein [41, 42]. As demonstrated by mass spectrometry (LC-MS/MS) [9, 43], the composition of the carbohydrate chains attached to the N- and O-glycosylation sites varied depending on the cell type used for the virus cultivation and differed in the native and recombinant protein [9]. The branching of the chains was even higher than originally predicted [12]. Five of the N-linked glycans stay exposed to the medium, even in the post-fusion spike conformation (which turned out to play no significant role in the virus evasion of the host immune system) [44].

**Fig. 3.**
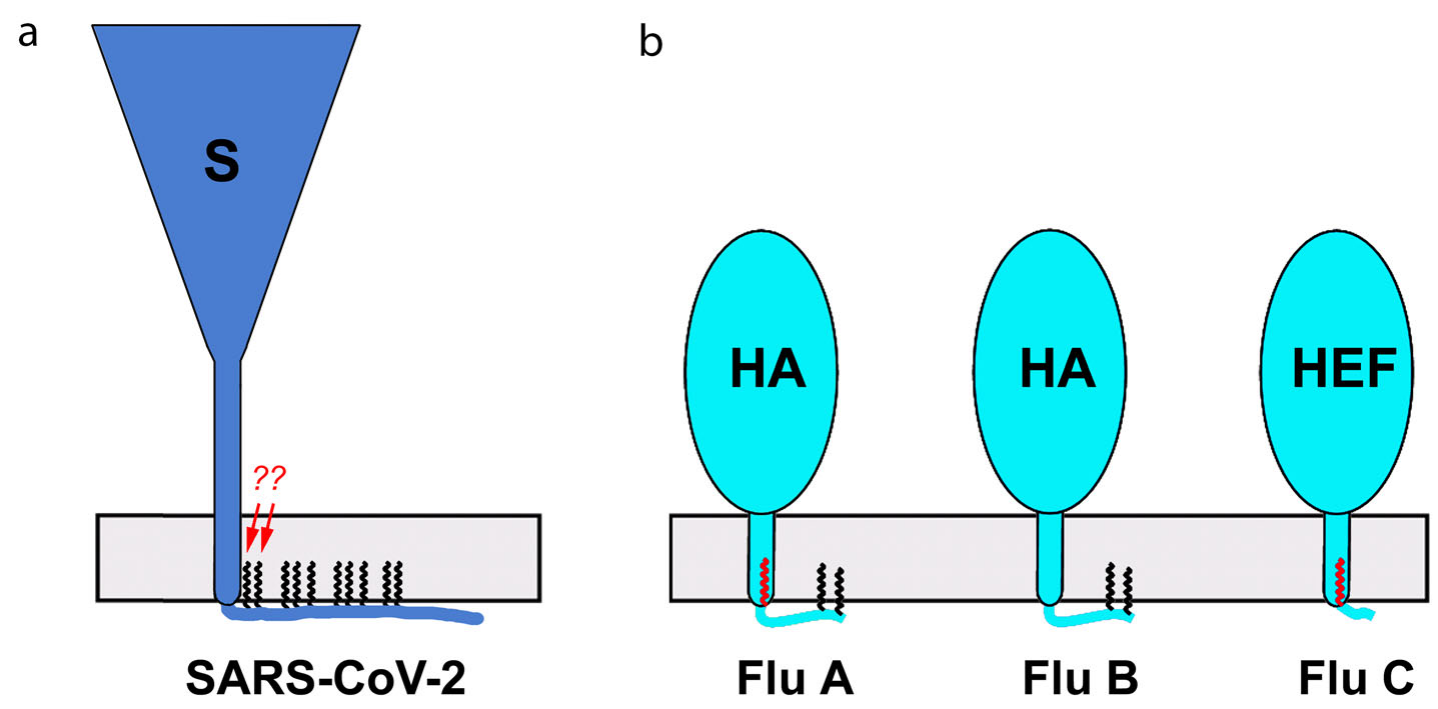
Modification of SARS‑CoV‑2 S protein (a) and HA/HEF from the influenza A, B, and C viruses (b) with long fatty acids. Covalently bound fatty acid residues are shown for one monomer of the homotrimer spike as black (palmitates, C16:0) and red (stearates, C18:0) zigzag lines. Two fatty acid residues that hypothetically bind stearates are marked with arrows. HAs and S protein are shown approximately to scale (S protein spike height, ~25 nm; [32]; HA spike height, ~13.5 nm [47]).

In addition to being extensive glycosylated (which affects the antigenic properties), S protein is S-palmitoylated (S-acylated) by long fatty acids at the conserved cysteine residues. This modification is involved in the processes of membrane fusion and virion assembly. A monomer of SARS‑CoV‑2 S protein contains a cluster of 10 S-acylated cysteine residues (30 residues in the homotrimer) in the 39-aa intraviral segment [21] (Fig. 3a). Fatty acid residues stabilize S protein and drive the formation of localized ordered lipid nanodomains enriched with sphingomyelins and cholesterol (typical composition of lipid rafts) already in the ERGIC, where maturation of the progeny virions takes place. In uninfected cell, ERGIC does not contain high cholesterol concentrations [21]. The substitution of cysteine residues in the CT domain with alanines results in dramatic changes in the S protein properties and decreases virus overall infectivity [21]. Two cysteine residues closest to the lipid membrane were found to be most functionally significant. It was suggested that their modification facilitates the binding of fatty acids to the C-terminal cysteine residues located farther away from the membrane, because this reaction is catalyzed by the integral membrane enzyme belonged to the family of ZDHHC acyltransferases [21]. This lipid modification is also typical for other coronaviruses, in which the number of S-acylation sites can vary from 6 to 10. It was found that modification with fatty acids is essential for the formation of functional virions [21, 45, 46].

It was shown previously that deletion of all three acylation sites in the influenza A virus HA suppressed virus reproduction, while deletion of one or two sites closest to the molecule C-terminus attenuated the virus [48]. We demonstrated using MALDI-TOF mass spectrometry that HAs from the three types of influenza virus (A, B, and C), as well as glycoproteins from some other enveloped viruses, are differentially S-acylated with two types of fatty acids. The stearate residue (C18:0) could be attached only to the cysteine residue located between the TM and CT domains, while cysteine residues in the CT domain bind exclusively palmitate residues (C16:0) [49-52] (Fig. 3b). Whether this pattern is preserved in the coronavirus S protein remains to be investigated.

**3D-Structure of the spike and its interaction with ACE2.** By the end of March 2021, more than a hundred of 3D-structures of the SARS‑CoV‑2 S protein had been submitted to the PDB, including the structures of the water-soluble ectodomain (both free and in complex with the ACE2 receptor), the full-size recombinant protein, and the spikes on the virion surface. Several variants of the pre-fusion and one version of the post-fusion 3D structures have been reported. To stabilize the pre-fusion structure of the isolated S protein, two proline residues were inserted in the S2 subunit after the HR1 sequence at positions 986 and 987; the polybasic furin cleavage site was removed and the TM and CT domains were replaced with the artificial domain (foldon, trimerization domain) [11, 53].

The key feature of the 3D structure of the S protein ectodomain in the pre-fusion conformation is the mobility of its RBD, which allows the switch between the closed (“all RBDs down”) conformation of the spike important for evading the neutralizing antibodies [54] and the open (“one RBD up”) conformation that initiates the binding to the ACE2 molecule followed by a cascade of S protein structural rearrangements, and, finally, membrane fusion. A remarkably wide variety of the pre-fusion structures have been described for a mixture of the S protein ectodomain and water-soluble ACE2 fragment [55]. Nine different conformations of the homotrimeric spike with RBDs in various positions, both before and after binding with the ACE2 receptor, have been reported (including S protein ectodomain with two open and one closed RBDs, although the fraction of such structures was insignificant) [55]. The complex of the S1 subunit with ACE2 was also detected.

The following hypothetic scheme for the interaction of the surface-exposed homotrimeric S protein spike with cell receptors was proposed. To initiate the binding, at least one RBD should be in the up position. This RBD binds to the peptidase domain of the ACE2 molecule, which initiates the opening of the RBD of the neighboring monomer. The binding of the latter to the second ACE2 receptor is followed by the opening and binding of the third RBD. Sequential binding of RBDs from all three S protein monomers with the peptidase domains of ACE2 molecules eventually results in the shedding of the S1 subunits, opening of the homotrimeric complex, and exposure of the S2′ sites of the S2 subunits, which are otherwise deeply hidden inside the spike. This makes the protein molecule accessible for the membrane TMPRSS2 protease that cleaves the polypeptide chain at this site [55] and releases the IFP (Fig. 2d).

Subsequent structural transition from the pre-fusion conformation to the post-fusion one results in the superposition of the fusion peptide and the TM domain at one end of the long structure centered around the three-stranded coiled coil [44]. Although the mechanism of membrane fusion during SARS‑CoV‑2 entry has not been investigated in detail yet, by analogy with other viruses using type I fusion proteins for the cell entry, it can be suggested that penetration of the fusion peptide into the target membrane promotes the formation of membrane pore, which then expands and allows the viral nucleocapsid to enter the cell cytoplasm [56].

**Co-receptors and antagonists.** Other S glycoprotein-binding determinants have been identified on the cell surface, which could facilitate the development of effective antiviral therapies. It was shown that the SARS‑CoV‑2 S protein interacts with heparan sulfate via the RBD, which promotes formation of the open spike conformation and enhances protein binding with ACE2 [57]. Therefore, heparan sulfate can be considered as a co-receptor (host attachment factor). This phenomenon was not observed for other human coronaviruses, such as SARS‑CoV‑1 and MERS‑CoV [57].

Linoleic acid (C18:2, essential fatty acid) was recently identified as an antagonist of SARS‑CoV‑2 binding to the cell membrane [58]. It was demonstrated using cryo-EM that the RBDs of the spike bind free linoleic acid in three composite binding pockets. Interestingly, in the S protein with bound linoleic acid, the RBM is highly ordered and hidden at the interface between the RBDs, while in the previously described cryo-EM structures, in particular, in the S protein complex with ACE2, the RBM was disordered [11, 12]. The binding of linoleic acid stabilizes the closed conformation of S protein, which reduces virus interaction with the ACE2 receptor *in vitro* [58]. It should be mentioned that in human cells, linoleic acid supplementation synergizes with the drug remdesivir [58].

**Fig. 4.**
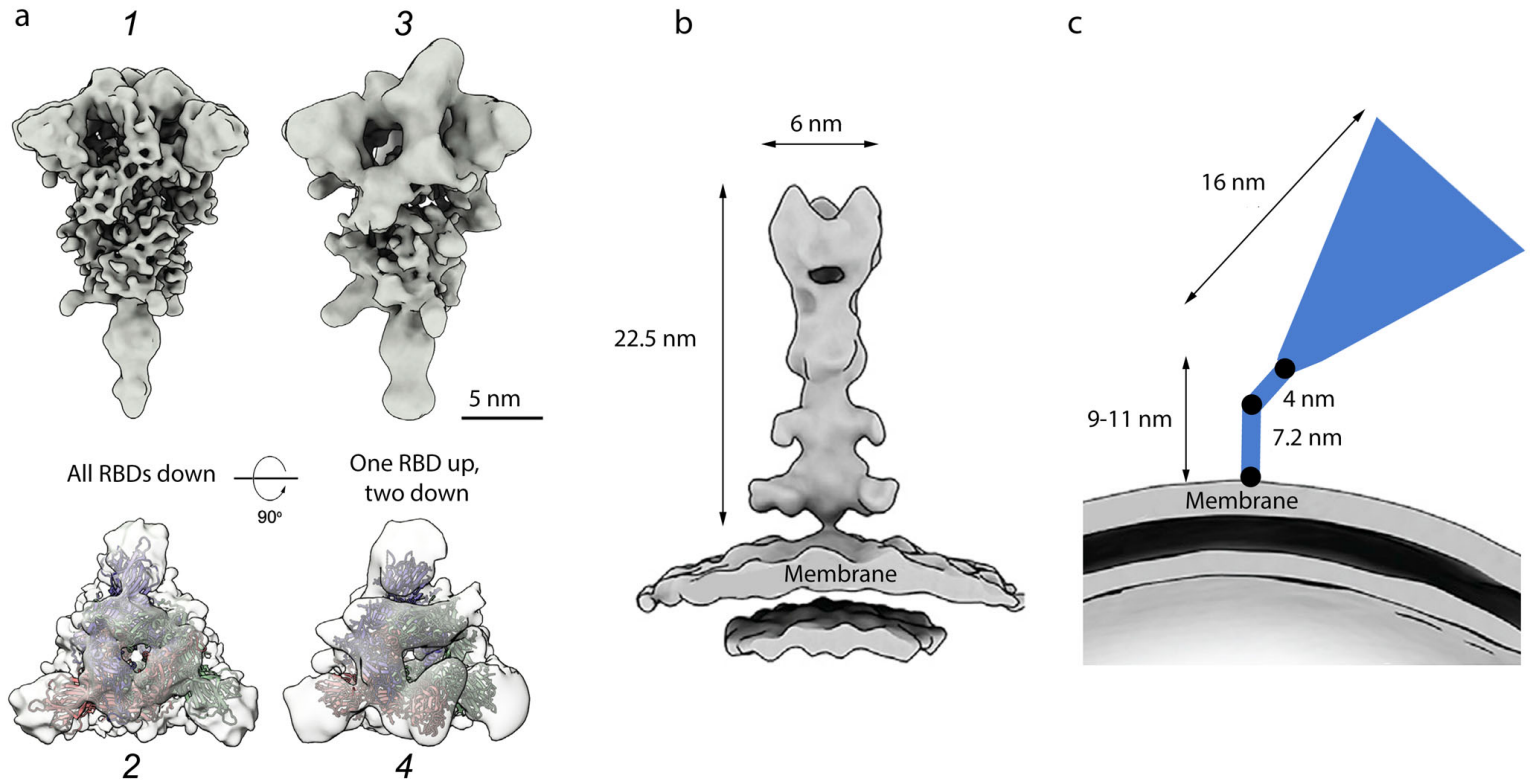
Cryo-EM structures of S protein homotrimeric spike on the surface of SARS‑CoV‑2 virions. a) Pre-fusion conformation: *1* and *2* – closed conformation (flail-like shape; all RBDs down); *3* and *4* – open conformation (one RBD up, two RBDs down); shown from the side (*1* and *3*) and top (*2* and *4*); b) post-fusion conformation (needle-like); c) tilted spike due to the flexible hinges in the stem (black circles) [41]. The spikes are shown to the scale according to [9, 16, 41]. Adapted with permission from Yao et al. [9]. Copyright Elsevier, 2020.

**Spikes topography on the virion surface.** The pre- and post-fusion conformations of the spike on the virion surface are remarkably different, which facilitates their identification even by the low-resolution cryo-EM (Fig. 4). While the spike in the pre-fusion state has the flail shape (Fig. 4a), it is narrower and has a needle-like shape in the post-fusion state (Fig. 4b).

The virion carries on average 24 ± 9 S protein trimers [10] (~40 trimers in [41] or ~5-60 trimers in [9]). In the virions fixed with formalin (saturated formaldehyde solution that cross-links proteins), approximately 97% spikes were found to be in the pre-fusion conformation and 3% – in the post-fusion conformation [10]. The spikes in each conformation did not form clusters but were distributed evenly on the virion’s surface. It is worth mentioning that when the viral particles were concentrated by a standard ultracentrifugation protocol, only spikes in the closed pre-fusion conformation were observed, while clarification of the virus-containing medium by the low-speed centrifugation resulted in the detection of both closed and open pre-fusion conformations of the spikes [10]. It is likely that the fragile open conformation does not survive ultracentrifugation.

Inactivation of virions with β-propiolactone, which binds to the viral nucleic acid, dramatically changed the ratio between the pre- and post-fusion S trimers: the fraction of spikes in the pre-fusion conformation was only 24% versus 76% spikes in the post-fusion conformation [59]. Cell entry of virions carrying on their surface a large number of spikes in the post-fusion conformation is significantly attenuated. When used as components of inactivated vaccines, such virions will most likely induce generation of either non-neutralizing or sub-neutralizing antibodies [60] that would be inefficient against a live virus. In the worst-case scenario, these antibodies will facilitate the development of the antibody-dependent enhancement (ADE) of infection upon the native virus entry [59].

Cryo-ET analysis revealed that the majority of S protein spikes are highly tilted towards the virion membrane (average angle, ~50° relative to the perpendicular position, reaching 90° in some cases) [10, 41] (Fig. 4c). Tilting of the spikes in all directions could be due to the presence of several flexible hinges in the long (9-11 nm) thin spike stem [41]. Such high flexibility of the spike could help the viral particle to scan the surface of the host cell for the most suitable binding sites. For comparison, the stem of the influenza virus HA homotrimeric spike is significantly shorter (1.8-2 nm) [47]. The flexibility of the HA trimers was reported previously [61, 62]; however, they are less flexible in comparison with the SARS‑CoV‑2 S trimers.

**S protein mutation rates.** The emergence of new genetic lineages and variants of the virus causes a serious concern, especially in the production of vaccines aimed against specific antigenic determinants of S protein. In particular, the D614G mutation, which is present in all SARS‑CoV‑2 lineages [63] and abolishes formation of the salt bridge with K854 [10], has increased the virus reproduction activity and infectivity rate (i.e., basic reproduction number, R0), which has likely become the root cause of the current pandemic [63-65]. The N501Y mutation in the S protein epitope resulted in emergence of a new genetic variant with the enhanced affinity to ACE2. At the time of writing this review, several virus variants with an increased infectivity rate have been identified that cause more severe disease. They include (i) 501Y.V1 or British Variant of Concern-202012/01, which is 50% more virulent; (ii) 501Y.V2 South Africa variant implicated in the decreased vaccine efficiency; (iii) 501Y.V3 Brazil variant with the mutation profile close to that of the South Africa variant. The genetic lineage B.1.351, which includes the 501Y.V2 variant, carries the E484K and K417N mutations in addition to N501Y. All three mutations are located in the RBD [66] (N501Y and E484K – in the RBM) and could potentially affect the affinity of the virus to the ACE2 receptor. The P681H mutation in the B.1.1.7 lineage located in close vicinity to the furin cleavage site could also be important in the context of virus infectivity (https://virological.org/t/preliminary-genomic-characterisation-of-an-emergent-SARS-CoV-2-lineage-in-the-uk-defined-by-a-novel-set-of-spike-mutations/563). A possible correlation between the emerging mutations and vaccine efficiency have been studied in [67]. In particular, it was shown that the E484K + N501Y + D614G group of mutations does not significantly reduce the ability of the antibodies produced in response to the BTN162b2 vaccine to neutralize this virus [67].

The information on the emerging mutations and genetic variants of SARS‑CoV‑2 is available on The New York Times website (https://www.nytimes.com/interactive/2021/health/coronavirus-variant-tracker.html), as well as in the global phylogenetic database GISAID (www.gisaid.org). Analysis of rapidly changing virus variants suggests that the variability of SARS‑CoV‑2 could in the nearest future come close to the variability of seasonal flu virus, which would direct the strategy of vaccine production towards predominantly polyvalent vaccines.

## COVID‑19 pathogenesis

Development of COVID‑19 in an infected individual occurs in three stages [68]. Stage I is initial binding of the virus to the ACE2 receptors of the respiratory epithelium in the upper respiratory tract followed by the virus replication with the help of TMPRSS2. At this stage, the virus can be detected in the nasal specimens by PCR. Stage I is characterized by the low viral load and weak immune response. Stage II involves the release of proinflammatory cytokines, interferons (IFNs) β and γ, by the epithelial cells. Stage III is characterized by the high viral load, hyperinflammation, and apoptosis of epithelial cells; the virus reaches lung alveoli. Finally, acute respiratory distress syndrome (ARDS) develops, characterized by the systemic organ dysfunction [68] accompanied by the reduction in the number of lymphocytes in the peripheral blood (lymphopenia) [69].

The accumulated observations indicate that the COVID‑19 progression is driven by the dysregulated and uncontrolled innate immune response. Activation of the pulmonary capillary endothelium results in the expression of cytokines and vascular cell adhesion molecules, which could exacerbate the cytokine storm and promote vascular thrombosis. Interleukin‑6 (IL‑6) and tumor necrosis factor (TNF) must be mentioned among the proinflammatory cytokines, the production of which could result in the epithelium dysfunction [1]. IL‑6 promotes vascular permeability and secretion of proinflammatory cytokines by the endothelial cells themselves, further upregulating the release of cytokines [1].

In severe COVID‑19 cases, massive endothelial dysfunction, extensive coagulopathy, and thrombosis caused by the complement system functioning could result in the development of systemic microangiopathy and thromboembolism. These complications are life threatening and could cause multiple organ dysfunction, including myocarditis, heart failure, pulmonary edema, hypoxia, and kidney damage [1]. Disruption of kidney functions is associated with the increased risk of mortality in seriously ill patients [1]. Individuals with diabetes and chronic obstructive pulmonary disease (COPD), i.e., diseases accompanied by the upregulated expression of ACE2 receptors, are more likely to develop complications during COVID‑19 [68]. Old age and vitamin D deficiency are also among the risk factors [70]. The studies in the animal models have revealed the hormone-mediated upregulation of ACE2 expression in males [70], which indicates the possibility of more severe course of COVID‑19 in men.

The central immunity paradigm states that the IFN-mediated anti-viral response occurs prior to the pro-inflammatory reactions, thus optimizing host protection and minimizing collateral damage [71]. This paradigm likely does not apply to COVID‑19. Studying the time patterns of IFN and inflammatory cytokines in 32 moderate-to-severe patients hospitalized with pneumonia and followed for the development of respiratory failure showed that the production of IFN‑λ and type I IFN was decreased and delayed, induced only in some patients who have become critically ill. On the contrary, the proinflammatory cytokines TNF, IL‑6, and IL‑8 were produced before IFNs in all patients and persisted for a long period of time. Higher IFN‑λ concentrations in the COVID‑19 patients correlated with the lower viral load in the bronchial aspirates and faster virus clearance. For comparison, both IFN‑λ and type I IFN were reliably induced to higher levels irrespectively of the disease severity in 16 flu patients hospitalized with pneumonia with similar clinicopathological symptoms as in COVID‑19 patients and in 24 non-hospitalized patients with mild flu symptoms, while the pro-inflammatory cytokines were produced only acutely. The altered cytokine patterns in the COVID‑19 patients correlated with longer hospitalization and higher occurrence of critical illness and death in comparison with the flu. These data indicate a dysregulation of the antiviral response in COVID‑19 patients, resulting in the persistent viral presence, hyperinflammation, and respiratory failure [72].

A wide range of serious pathological conditions, such as bilateral pneumonia, heart failure, and liver failure, have been reported for the outbreaks of A/H5N1 and A/H7N9 flu in humans and during the A/H1N1 pandemic in 2009 [14]. New data indicate that the endothelial dysfunction induced by the SARS‑CoV‑2 infection differs from the effects produced by the 2009 influenza pandemic A/H1N1 strains. For instance, COVID‑19 infection can cause pyroptosis which can lead to the death of endothelial cells and promote proinflammatory stimuli and thrombotic events [73]. Obviously, clinical manifestations of severe flu illness differ from those typical for severe COVID‑19 cases (although certain similarities do exist, such as initiation of cytokine storm). As a rule, no serious immune system dysfunction is observed in seasonal flu.

Morphometric studies have demonstrated that the walls of the pulmonary arteries in the COVID‑19 patients are twice as thick (hence, the lumen diameter is smaller) than in the patients infected with the 2009 pandemic influenza H1N1 virus [74]. New data are emerging that the S protein itself could trigger signaling cascades harmful for the cells [74], thus facilitating development of a serious pathological state called pulmonary arterial hypertension [75]. Considering that the SARS‑CoV‑2 S protein is the main antigenic component of vaccines, we believe that it is crucial to investigate further its potential effects on the cells of pulmonary arteries and other organs, such as systemic vessels, heart, and brain [75].

## COVID‑19 THERAPY

**Potential antiviral preparations and therapeutic antibodies.** The antiviral preparations targeted at other viral infections can be hypothetically repurposed to fight COVID‑19 [76, 77]. The key protein components of SARS‑CoV‑2 that have been used as targets for antiviral drugs are the S protein, RNA-dependent RNA-polymerase (RdRp), and viral protease Mpro. The most known among such drugs is Remdesivir, a synthetic analogue of adenosine, which binds RdRp and blocks the synthesis of viral RNA [78]. Although this drug developed by Gilead Sciences (USA) demonstrated no differences from placebo in the treatment of moderate-to-severe COVID‑19 [79], administration of Remdesivir at the early stages of COVID‑19 infection provided some benefits. Remdesivir has been approved for emergency use in 50 countries (https://www.covid19treatmentguidelines.nih.gov/therapeutic-management/).

Camostat mesylate [80] and Nafamostat [81] were found to inhibit membrane TMPRSS2; other commercially available preparations (Talampicillin, Lurasidone, Rubitecan, Loprazolam) [82] may also affect cellular proteases. Triazavirin exhibiting the antiviral activity against different viruses is a potential inhibitor of Mpro [83-85]. The effects of other preparations declared as antiviral (Favipiravir, Ivermectin, Ribavirin) on SARS‑CoV‑2 are currently being investigated [64]. Some drug candidates (Lopinavir, Ritonavir, Hydroxychloroquine) have been recognized as ineffective.

Several promising antiviral strategies have been suggested by authors of numerous fundamental studies; however, such strategies are still mostly ideas. In particular, a synthetic lipopeptide with the amino acid sequence of the HR2 domain able to block the conformational rearrangements in S protein, was suggested to suppress the fusion of the viral and cellular membranes [86]. Another approach is the knockdown of ZDHHC20 acyltransferase that acylates S protein in order to reduce reproduction of viral particles and their infectivity [21]. As direct administration of type I IFN preparations used to limit the infection can produce some negative effects, a new strategy was proposed that involves activation of the interferon-stimulated genes (ISGs) encoding, in particular, IFN-induced transmembrane proteins (IFITMs) and cholesterol 25-hydroxylase (CH25H) [87].

The possibility of the inhibition of the S protein binding to ACE2 by exogenous heparin and its non-anticoagulant derivatives opens new therapeutic possibilities [57]. Thus, drugs interfering with the virus binding to heparan sulfate on the cell surface have been suggested for the use in the combination antiviral therapy [57, 88, 89].

Neutralizing antibodies were among the first antiviral preparations used in the COVID‑19 pandemic [12, 90, 91]. Monoclonal antibodies isolated from the convalescent patients exhibited the neutralizing capacity by blocking the contacts between the S protein RBD and ACE2 [91]. However, the emergence of mutations in the RBD can hinder the antibody-based therapy [92]. Administration of a noncompeting pair of antibodies recognizing different RBD epitopes enhanced the blocking effect of the used preparations in clinical application and helped to minimize the possibility of the virus escaping the immune response [91].

**Blocking the pathogenetic processes.** One year since the start of the pandemic, it has become clear that the majority of antiviral preparation have so far failed to improve the clinical outcomes of severe COVID‑19 cases. On the contrary, therapeutic interventions targeting the pathologic responses of the patient’s organism (hyperimmune response, complement activation, and systemic thrombosis) have been found as more promising [1]. The following preparations are used in the clinical practice in Russia:

- *Dexamethasone*, a synthetic glucocorticoid used for the treatment of rheumatic diseases, some skin diseases, severe allergic reactions, asthma, COPD, cerebral edema, as well as tuberculosis in combination with antibiotics [2, 64].
- *Olokizumab*, a preparation of monoclonal antibodies inhibiting IL‑6. It was previously developed by UCB Pharma (Belgium) for the treatment of rheumatoid arthritis [93] and is now produced by R‑Farma (Russia).
- *Anti-inflammatory antibodies in combination with anticoagulants* used to limit coagulopathies and abnormal cytokine signaling initiated by SARS‑CoV‑2 [94].

## PRINCIPLES OF PROPHYLACTIC IMMUNIZATION

At present, prophylactic vaccines represent the only strategy capable of limiting the spread of the coronavirus infection. As of February 2021, 289 candidate vaccines against SARS‑CoV‑2 have been reported and around 70 of them were in clinical trials (https://www.who.int/teams/blueprint/COVID-19). We are witnessing a new worldwide technological revolution in vaccine production [95]. The vaccines approved for the use against SARS‑CoV‑2 include both traditional inactivated and recombinant vaccines, as well as innovative preparations based on the delivery of genetic material coding for the target antigen [53]. The new vaccines use viral DNA or RNA to reproduce the natural processes of transcription, translation, and expression of cellular proteins.

**RNA-based vaccines.** Different RNA variants have been tested over the years for the development of effective RNA vaccine that would still retain several core elements present in the intracellular mRNA, such as the cap structure (7-methyl guanosine), 5′-UTR, sequence coding for the target protein (SARS‑CoV‑2 S protein in the case of COVID‑19), 3′-UTR, and polyadenylation site. Administration of an RNA vaccine is followed by a series of cellular events leading eventually to the immune response development. These events can be divided into three steps, each requiring certain approaches and optimization procedures: (i) delivery of RNA into the cell and overcoming the cell barrier; (ii) induction of IFNs, or self-adjuvant effect; and (iii) antigen processing for the major histocompatibility complexes class I and II (MHC‑I and MHC‑II).

RNA protection from the degradation with RNases in the extracellular space and blood flow is very important at the first step of mRNA delivery through the plasma membrane, as it determines the efficiency of this delivery. RNA is negatively charged and cannot passively diffuse through the membrane, thus requiring the participation of the active transport systems. To solve this problem, lipoplexes (complexes of nucleic acids with lipids) that can enter the cell via endocytosis, have been suggested [96-100]. The mixtures used for the mRNA delivery usually consist of cationic ionizable lipids and lipids for the structure stabilization, e.g., phospholipids, cholesterol, or polyethylene glycol-modified lipids (PEG-lipids). Cationic lipids are added to ensure formation of complexes with the negatively charged mRNA. Cationic lipids are classified based on the *p*K of the lipid amino group as pH-dependent ionizable lipids and lipids with a constant charge, such as DOTMA (1,2-di-O-octadecenyl-3-trimethylammonium propane), DOTAP (1,2-dioleyloxy-3-trimethylammonium propane), DC-cholesterol [3β-N-(N′,N′-dimethylaminoethane)-carbamoylcholesterol]. The former carry a positive charge at low pH, which facilitates formation of complexes with RNA molecules. The charge of the complexes varies from neutral to slightly positive under physiological pH, which reduces their toxicity, prevents nonspecific interactions with serum proteins, and prolongs their blood circulation time [101, 102].

**Fig. 5.**
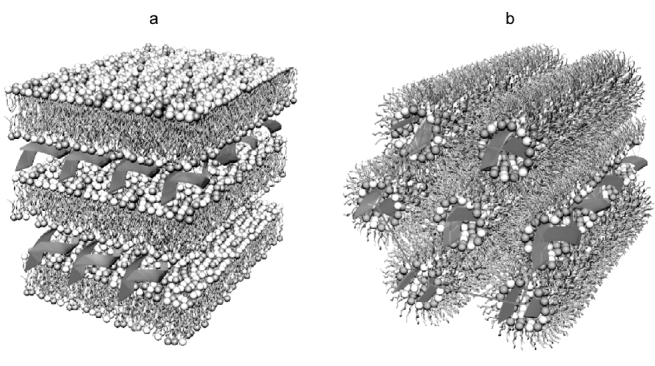
Structural variants of lipoplexes: a) multilayer lamellar structure; b) inverted hexagonal structure. Adapted with permission from the review by Ewert et al. [109]. Copyright Taylor & Francis, 2005.

The lipofection technology for intradermal injections has been described in numerous papers including those using mRNA for vaccination against influenza A/H10N8 and A/H7N9 viruses, Dengue virus, as well as for the treatment of type II diabetes [103-105]. This technology is based on the preparation of lipid nanoparticles with a size of 80-120 nm. To produce lipoplexes, lipids are dissolved in alcohol at a certain ratio and mixed with the RNA solution in a buffer with low pH (~4.0) using a microfluidic mixer [98]. The structure of the lipoplex particles is diverse and can vary from lamellar to spherical and hexagonal, although the distribution of the DNA/RNA strands and lipid bilayers remains the same (Fig. 5). Moderna (USA) is a pioneer in developing novel mRNA-based vaccine against SARS‑CoV‑2. This company has been also developing prototypes of mRNA vaccines against Zika virus [98, 106], influenza virus [107], and RSV [108].

The main targets of vaccination are antigen-presenting cells, e.g., dendritic cells (DCs), since they represent a link between the antigen reception and development of T-cell and B-cell immune response. DCs present antigens after taking them up from either the cytoplasm, which involves presentation of the antigenic peptides by the MHC I, or lysosomes that carry the antigen fragments taken up by endocytosis (presentation via MHC II). MHC I and II are recognized by the receptors of CD8^+^ or CD4^+^ T cells, respectively.

Induction of IFNs, or the self-adjuvant effect, is the main trigger of a series of immunomodulating effects caused by the RNA vaccine after its entry to the cell that can have either positive or negative consequences. In general, acute action of type I IFN mediates the pleiotropic and inflammatory effects in both innate and adaptive immune responses [110, 111]. Type I IFN signaling induces maturation of DCs, promotes antigen processing and presentation, and facilitates migration of DCs to the areas of transfected cells [112]. However, it can also lead to the development of severe autoimmune disorders, such as lupus or type I diabetes [113, 114]. Specific structural features of mRNA used in the vaccines allow one to overcome this problem via balancing expression of the target proteins and INF-mediated inflammation and autoimmune reactions.

The first mRNA vaccine against the new coronavirus infection was the BNT162b2 vaccine developed by the German company BioNTech and American company Pfizer. The results of clinical trials involving 43,998 patients showed that the efficacy of BNT162b2 vaccine was ~95% (ClinicalTrials.Gov/NCT04368728, Pfizer/BioNTech, https://clinicaltrials.gov/ct2/show/NCT04368728). The second mRNA vaccine (mRNA‑1273) was developed by Moderna in collaboration with the National Institutes of Health (USA). The results of phase III clinical trials involving 28,207 patients demonstrated that the efficacy of this new vaccine was 94.5% (ClinicalTrials.Gov/NCT04470427, Moderna, https://clinicaltrials.gov/ct2/show/NCT04470427). Both RNA vaccines require 2 intramuscular injections with a 3- to 4-week interval between the doses; the vaccines must be transported at temperatures from –25°C to –15°C, while –80°C is strictly recommended for periods of storage exceeding two weeks (Pfizer-BioNTech COVID‑19 Vaccine Storage and Handling Summary, https://www.cdc.gov).

**Adenovirus-based (Ad-vectored) vaccines.** Modified adenoviruses have been widely used as vehicles for the intracellular delivery of genetic material and development of vaccine prototypes since the discovery of the possibility of their application in genetic engineering in the early 1990s [115]. A DNA sequence encoding the target protein (SARS‑CoV‑2 S protein in the case of COVID‑19) is inserted in the adenoviral genome using molecular cloning techniques in order to induce the immune response. This technology uses modified replication-defective adenoviruses (vectors) based on rare species or types of viruses absent in the human population, e.g., human virus Ad26 or viruses of chimpanzees and non-human primates, to avoid possible pre-existing immunity to adenoviruses. In the case of adenoviral vectors, the target protein is expressed in the host cells for two weeks on average, with the peak expression on days 2-3, which is sufficient for the immune response development [116].

Modern replication-defective adenoviruses can propagate only in special cell lines, such as HEK 293 or PER.C6 (packaging cell line) that carry the entire 5′-terminal part of the viral genome, including sequences for E1 and protein IX, inverted terminal repeats (ITRs), and *cis*-acting packaging sequences. Cloning of adenoviral vectors as components of plasmids allows their manipulation and propagation in *Escherichia coli* cells, while the use of a strong cytomegaloviral promoter provides high expression levels of target genes in comparison with other genetic engineering techniques for the intracellular delivery of coding sequences [117].

Adenovirus particle unpacks upon its entry into the host cell. The viral DNA is transferred to the nucleus via the microtubules, where it serves as a template for self-replication. Considering that the life cycle of a wild-type adenovirus is extrachromosomal, the Ad-vectors are assumed to be non-integrating. However, the recombination between the vector and chromosomal DNA has been demonstrated in a number of model experiments in mice [118]. This phenomenon should be investigated further to elucidate possible long-term effects and safety of the vector vaccines.

Immune response to the adenoviral particles itself attracts a close attention of many researchers. Numerous data exist confirming a significant reduction in the generation of antibodies against the target protein during repeated immunization with the same vector [119], which is a serious obstacle for the use of adenoviral vectors in the development of vaccination strategies, especially considering that COVID‑19 might become a seasonal disease with constantly changing circulating variants.

At the time of writing this review, several vaccines based on the adenovirus-mediated delivery of genetic material have been approved and released on the market worldwide. The two-dose vaccine Sputnik V registered in Russia under the name Gam‑COVID‑vac uses a heterologous system for the target gene (SARS‑CoV‑2 S protein) delivery based on the two types of adenoviruses, Ad26 and Ad5. This vaccine demonstrated 91.6% efficacy against COVID‑19 and safety in phase III clinical trials involving more than 16,500 participants [120].

The AZD1222 vaccine developed and released by the AstraZeneka company in collaboration with the Oxford University is based on the chimpanzee adenoviral vector ChAdOx1. The vaccine efficacy (79% against SARS‑CoV‑2 infection and 100% against severe forms of COVID‑19) was demonstrated in phase II clinical trials (ClinicalTrials.gov/NCT04516746, AstraZeneca, AZD1222, https://clinicaltrials.gov/ct2/show/NCT04516746?term=NCT04516746&draw=2&rank=1). A single-dose vaccine based on the Ad26 vector (61% to 72% efficacy depending on the country where the trials were conducted) has been developed and released by the Janssen Biotech, Inc. CanSino Biologics also developed a single-dose vaccine based on the Ad5 vector that demonstrated 65.28% efficacy.

**Inactivated whole-virion and split vaccines.** Another type of vaccines against SARS‑CoV‑2 that has been developed and approved for the use by regional authorities, are inactivated whole-virion and split vaccines. In the latter (improved) type, inactivated virions are additionally destroyed by detergents, such as Triton X‑100. Several preparations produced by Sinopharm, Sinovac, and Chumakov Federal Scientific Center for Research and Development of Immune and Biological Products (Russian Academy of Sciences) have been approved for immunization of limited groups of people. Despite insignificant differences in technological processes, production of such vaccines requires special attention to the conformation of S protein in the final preparations, taking into account inactivation of the live virus by β-propiolactone, which may promote transition of S protein pre-fusion conformation to post-fusion one (as mentioned above). An uncontrolled use of inactivating agents could result in the induction of non-neutralizing antibodies in the host organism.

**Peptide vaccines.** The first synthetic peptide vaccine EpiVacCorona against the new coronavirus was developed by the VECTOR Center of Virology, Rospotrebnadzor. It is a suspension for intramuscular administration that contains a mixture of chemically synthesized peptide immunogens of the SARS‑CoV‑2 S protein conjugated with the protein carrier and adsorbed onto aluminum hydroxide. The first data on its application were published recently [121]. Currently, this vaccine is in phase I-II clinical trials. It has been stated that the peptide-based EpiVacCorona vaccine exhibits immunological activity, safety, and low reactogenicity. Further studies are needed to determine its efficacy.

## CONCLUSIONS

One year ago, a sudden emergence and rapid spread of the COVID‑19 pandemic have given rise to numerous speculations and rumors. Currently, the experts consider the possibility of SARS‑CoV‑2 origin as a result of laboratory manipulations with a related coronavirus as highly unlikely [122]. Genetic data provide strong evidence that SARS‑CoV‑2 is not a derivative of some previously used viral backbone. The WHO experts have come to the same conclusion in the report on the investigation by special commission in China published on March 30, 2021 (https://www.who.int/publications/i/item/who-convened-global-study-of-origins-of-SARS-CoV-2-china-part). The prevailing hypothesis on the origin of the new coronavirus leading to the pandemic is zoonosis, i.e., virus transmission from animals to humans.

A massive amount of data on the SARS‑CoV‑2 spike protein have been accumulated over the year 2020. The variety of the obtained 3D-structures have allowed to identify several key features of S protein that determine the specifics of the SARS‑CoV‑2 pathogenesis: (i) closed pre-fusion conformation of the spike could be used by the virus to evade host immunity; (ii) high affinity of the RBD open conformation to the human ACE2 receptor is important for efficient virus binding to the cell surface; (iii) preliminary activation of S protein by furin-like proteases facilitates virus propagation in different types of cells. These data have formed a basis for the development of antiviral preparations and vaccines. However, many aspects associated with the mechanism of virus replication, its damaging effects on an organism, and possible therapeutic strategies need further investigation.

The COVID‑19 pandemic has posed many new challenges to the global healthcare like no other disease before, resulting in introduction of new types of vaccines. Vaccination of 7 billion people requires an unprecedented globally coordinated effort. One of the key questions for the future is whether the T-cell immunity formed in the infected and vaccinated people would be strong enough. The data on the long-term effects of vaccination are still being accumulated. However, the early data on the new coronavirus patients are promising, as they demonstrate high levels of CD4^+^ and CD8^+^ memory T-cells against several SARS‑CoV‑2 proteins (in particular, nucleocapsid and membrane protein) beside the S protein [123]. Continuous monitoring of recovered patients should provide data on the protective capacity of both humoral and cellular immunity. It is important to understand how the T‑cell immunity is formed in patients with mild COVID‑19 symptoms in comparison with patients with severe forms of this disease.

To end on a positive note, there are reasons to believe that mutational variability will lead to the weakening of SARS‑CoV‑2. If this is the case, the virus will cause regular seasonal outbreaks of the disease with mild symptoms, similar to the seasonal flu, which will be less damaging for the humankind than the pandemic of 2020-2021.

## Abbreviations

aa: amino acid residue
ACE2: angiotensin-converting enzyme 2
COVID‑19: COronaVIrus Disease 2019
cryo-EM: cryogenic electron microscopy
cryo-ET: electron cryotomography
CT: cytoplasmic tail
ERGIC: endoplasmic reticulum-Golgi intermediate compartment
HA: hemagglutinin
IFN: interferon
MHC: major histocompatibility complex
RBD: receptor-binding domain
RBM: receptor-binding motif
SARS‑CoV‑2: severe acute respiratory syndrome coronavirus 2
TM: transmembrane
